# Insights into the role of internal catalysts in asparagine-to-succinimide conversion in a hyperthermophilic glutamine amidotransferase

**DOI:** 10.1016/j.jbc.2026.113093

**Published:** 2026-04-28

**Authors:** Anusha Chandrashekarmath, Oishika Jash, Karandeep Singh, Asutosh Bellur, Chitralekha Sen Roy, Aparna Dongre, Nayana Edavan Chathoth, Padmesh Anjukandi, Sanjeev Kumar, Souradip Mukherjee, Padmanabhan Balaram, Sundaram Balasubramanian, Hemalatha Balaram

**Affiliations:** 1 Molecular Biology and Genetics Unit, Jawaharlal Nehru Centre for Advanced Scientific Research, Bengaluru, India; 2 Chemistry and Physics of Materials Unit, Jawaharlal Nehru Centre for Advanced Scientific Research, Bengaluru, India; 3 Department of Chemistry, Indian Institute of Technology, Palakkad, Kerala, India; 4 National Centre for Biological Sciences, Tata Institute of Fundamental Research, Bengaluru, India

**Keywords:** crystal structure, glutamine amidotransferase, mass spectrometry, *Methanocaldococcus jannaschii*, protein thermostability, QM/MM MD simulation, succinimide

## Abstract

Succinimide (SNN), an intermediate spontaneously formed during asparaginyl deamidation or aspartyl dehydration in proteins, is generally hydrolysis-prone, leading to isomerization to an L/D α/β-aspartyl residue, with the latter being considered deleterious to protein structure and function. An unusually stable SNN-mediated conformational rigidity through restriction of the backbone dihedral angle, ψ, enhances the thermostability of glutamine amidotransferase (GATase) from *Methanocaldococcus jannaschii* (Mj). Although several structural features involved in maintaining a stable SNN and imparting SNN-mediated thermostability have been identified in MjGATase, the residues in the protein that catalyse the rapid and complete conversion of Asn109 to SNN remain unknown. Here, we investigated several mutants of MjGATase for their ability to retain the Asn109 side chain in the unmodified form. Mass spectrometric analysis of 10 single amino acid variants enabled the identification of residues that impacted the proportion of SNN and Asn population in the protein sample. This led to the generation of two double mutants of MjGATase that retained an intact Asn109 side chain, as confirmed by mass spectrometry and crystal structure analysis. These mutant proteins with intact Asn residue at position 109 displayed lower thermal stability than the protein with the SNN modification. Further understanding of the deprotonation mechanism was addressed using quantum mechanics/molecular mechanics molecular dynamics metadynamics simulations.

Deamidation, a largely spontaneous posttranslational modification (PTM) in proteins occurs at a higher rate in asparaginyl (Asn) compared to glutaminyl (Gln) residues (1, 2, 3). The process of Asn and Gln deamidation leading to Asp/iso-Asp and Glu/iso-Glu residues proceeds through the cyclic intermediates succinimide (SNN) and glutarimide, respectively (3, 4, 5). SNN is also an intermediate during aspartyl dehydration (6, 7). The mechanism of SNN formation during the deamidation of Asn residues proceeds in three main steps namely, (a) deprotonation of the backbone amide NH of the n+1 residue, (b) nucleophilic attack of the deprotonated amide nitrogen on the side chain carbonyl carbon of the Asn residue forming a tetrahedral intermediate, and (c) ammonia loss leading to SNN formation (8) (Fig. 1). The initial step of backbone amide deprotonation is a general-base catalyzed reaction, and the protonation of the leaving side chain NH_2_ group is a general-acid catalyzed reaction (8). The cyclic SNN formed is usually unstable and undergoes hydrolysis to an Asp/iso-Asp residue. Racemization of L-SNN to D-SNN followed by hydrolysis can also yield D-Asp/D-iso-Asp (6). Presence of SNN or its hydrolyzed products displays varied functional roles in peptides and proteins, including loss of function (9, 10, 11, 12, 13, 14, 15, 16, 17, 18, 19), alteration of *in vivo* turnover, regulation of time-dependent biological processes (20, 21, 22, 23), mediation of protein splicing (6, 24, 25, 26), and prevention of protein aggregation (27).

The rates of Asn deamidation are well studied in peptides, where it has been observed that the rate is influenced by several factors such as temperature, pH, ionic strength, and to a great extent, the nature of the succeeding residue (1, 6, 8, 28, 29, 30). However, deamidation rates in proteins do not necessarily follow the sequence pattern seen in peptides and instead are governed by the three-dimensional structure of the protein (31, 32, 33, 34). The deamidation rates of Asn residues examined in nearly 120 human proteins showed variation governed by both primary sequence and tertiary structure (35). The effect of three-dimensional structure on deamidation has been studied in a few proteins, such as RNase A (31) and trypsin (32), and by molecular dynamics (MD) simulations on the capsid protein of norovirus (36), and on triosephosphate isomerase (TPI), which includes a quantum mechanics/molecular mechanics (QM/MM) free energy calculation, providing a mechanistic understanding of the reaction pathway to the tetrahedral intermediate leading to SNN formation (37).

Our earlier mass spectrometry (MS) studies on *Methanocaldococcus jannaschii* glutamine amidotransferase (MjGATase) established the role of a remarkably stable SNN, arising from the deamidation of Asn109, in imparting hyperthermostability to the protein (38). MjGATase (UniProt ID: Q58970, E.C. 6.3.5.2), the glutamine amidotransferase subunit of an archaeal GMP synthetase (GMPS), hydrolyses glutamine, and the ammonia generated is channeled to the bound ATP pyrophosphatase (ATPPase; UniProt ID: Q58531) subunit, thereby catalyzing the conversion of xanthosine 5′-monophosphate (XMP) to GMP (39). Substitution of Asn109 with serine in MjGATase, thereby leading to abrogation of SNN formation, resulted in a protein with reduced thermal stability (38). Subsequent determination of MjGATase crystal structure enabled visualization of the SNN modification at residue 109 and its local structural environment (40). Structural analysis along with enhanced sampling MD simulations showed that the SNN induced a conformational lock, and together with long-range interactions, contributes to the enhanced thermal stability of the protein. The SNN itself is shielded from hydrolysis by the negative charge of the side chain carboxylate of the succeeding Asp110 residue and, through an n-π^∗^ interaction with the backbone C=O of the preceding residue, Glu108. These studies provided insights into the molecular basis of SNN’s resistance to hydrolysis and the role of this PTM in enhancing protein thermal stability (38, 40). However, the mechanism underlying the rapid and complete conversion of Asn109 to SNN in MjGATase remains to be explored. In addition, a search of the Protein Data Bank (PDB) yielded approximately 50 unique proteins containing stable SNN modifications; yet, a comprehensive understanding of the mechanisms governing SNN formation in these systems is lacking.

As the three-dimensional structure of the protein plays a major role in deamidation, it is imperative to understand the distinct structural features in MjGATase that may promote deamidation. In this study, from an analysis of the available structures of MjGATase and its mutants (40), we identified residues that could act as potential internal catalysts mediating SNN formation. These residues directly or indirectly contact the SNN within 4 Å distance cutoff. Ten single amino acid variants were generated and examined by MS for the presence of Asn109 with intact side chain. This analysis led to the identification of the residues Lys151, Asp110, and Tyr158, whose alterations in combination may completely abrogate SNN formation. The double mutants, D110V_K151L and K151L_Y158F, of MjGATase were found by MS to largely retain the intact Asn109 side chain, a finding that was validated by X-ray crystallography. MjGATase mutants harboring intact Asn109 exhibited reduced thermal stability confirming earlier observations (38) on the role of SNN in enhancing T_m_ values.

The dihedral angles ψ and χ1 of an Asn residue influence SNN formation (6), which requires a short distance between the CG of Asn and the backbone N atom of the (n+1) residue (3, 41). Apart from the dihedral angle and distance criterion, other structure based methods have been used to predict deamidation hotspots in antibodies and other proteins (34, 42, 43, 44). Using quantities obtained from detailed classical molecular dynamics (CMD) simulations such as the solvent accessible surface area of the aspartyl side chain, root mean square fluctuations of CA atoms of the aspartyl or Asn residue, solvent accessible surface area of the (n+1) N and H atoms, and solvent accessibility considering the local environment, the likelihood of degradation mediated through the SNN intermediate has been predicted (45, 46). In addition, the propensity of an Asn residue to undergo deamidation has also been examined through MD simulations combined with quantum mechanical calculations (47). From the perspective of a mechanistic understanding, there are only a few studies that discuss the deamidation reaction leading to SNN formation from a QM/MM perspective (37, 48, 49). Herein, we estimate the activation barrier for the deprotonation step of this PTM based on QM/MM MD simulations through the metadynamics method (50).

## Results

### Structure analysis suggests residues that may be implicated in the catalysis of SNN formation

The SNN atoms are numbered as shown in Figure 2*A*. The conversion of an Asn residue to SNN requires a base in the vicinity of the amide NH of the n+1 residue for proton abstraction. A search for a potential base capable of abstracting the amide NH proton of the n+1 residue (Asp110) was conducted to generate mutants of MjGATase that would be incapable of forming the SNN and hence retain an intact Asn side chain at residue 109. For this purpose, the contacts of the SNN atoms within a distance cutoff of 4 Å were identified in the structure of WT MjGATase (40) (Fig. 2, *B* and *C*, list in panel *D*). Henceforth, the WT MjGATase structure will be referred to as MjGATase_SNN_109_. An H-bonding interaction was observed between OD1 of SNN109 and Leu111 NH, in addition to contacts with Asp110 OD2, Lys151 CE, Leu139 CD1, Tyr158 CE2, and the CG of Leu111. The O109 of SNN contacts Glu108 carbonyl O and Lys113 CD. The backbone N of Asp110 (N2, Fig. 2*A*), which is part of the SNN109, contacts Asp110 OD1 and OD2, and Glu108 carbonyl O. Of the four carbons of the SNN109 ring, CB contacts Lys107 carbonyl O. Although the distance between the amide nitrogen atom (N1, Fig. 2*A*) of SNN109 and carbonyl O of Lys107 is 3.3 Å—suggesting a potential H-bond—the angle criterion for such an interaction does not appear to be satisfied (observed angle: 75°; accepted angle: >140°). In addition to these contacts with SNN109, we observed secondary H-bonding interactions between Lys151 NZ and Glu137 OE2 along with Glu137 OE1 and Lys107 NZ suggesting a possible proton relay.

**Figure 1:**
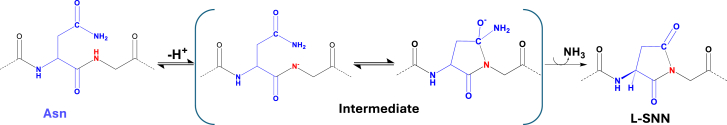
**Schematic of succinimide formation by an asparaginyl residue.** For the labeling of atoms in SNN, refer to Figure 2*A*. SNN, succinimide.

**Figure 2:**
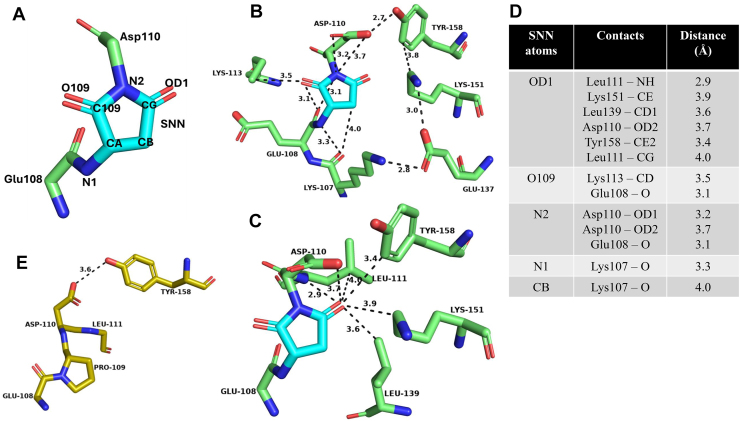
**Search for a potential base to abstract the n+1 amide NH proton.***A*, labeling of atoms in SNN. *B* and *C*, contacts of the succinimide within a 4 Å distance cutoff, in two different views in the MjGATase_SNN_109_ structure (PDB ID: 7D40). *D*, list of contacts made by atoms of SNN with atoms of other residues in MjGATase_SNN_109_. Contact distances in Å are provided. *E,* the contact between Asp110 side chain C=O and Tyr158 OH in MjGATase_N109P structure (PDB ID: 7D97). The number above the dashed lines in *panels* B, C, and E indicates distance in Å. MjGATase, glutamine amidotransferase from *Methanocaldococcus jannaschii*; SNN, succinimide.

The contact analysis described above suggests that the residues Asp110, Tyr158, Lys151, Glu137, Lys107, Glu108, and Lys113 could have direct or indirect roles in the abstraction of the Asp110 amide proton, thereby initiating SNN formation. Of these residues, as Asp110 side chain carboxylate group interacts with Asp110 NH (N2, Fig. 2, *A* and *B*), it appears as a possible potential base for abstracting the proton from its backbone NH. The Asp110 residue was changed to the imino acid, proline, that lacking the amide NH proton would be incapable of forming SNN and, therefore, retain the Asn109 side chain. Additionally, Asp110 was also substituted with Asn and Val to obliterate the negative charge, thereby eliminating the basic character of the side chain.

The second residue identified from the contact analysis as potentially assisting in proton abstraction by functioning as a component of a proton relay is Tyr158, the side chain OH of which contacts Asp110 carboxylate (Fig. 2*B*). Interestingly, even in the mutant, MjGATase_N109P (40), the H-bond between Asp110 carboxylate and Tyr158 side chain OH continues to be retained (Fig. 2*E*). Similarly, a proton relay could also occur through Lys151, Glu137, and Lys107 (Fig. 2*B*), hence, the mutants Y158F, K151L, E137L, and K107L were generated.

Although a direct contact between the side chain of Glu108 and SNN is not observed, this residue was changed to leucine and glutamine as it precedes SNN and could have implications in both the formation and stability of this PTM. The final residue identified from the contact analysis is Lys113, whose side chain CD is within a 4 Å distance cutoff from SNN (Fig. 2*B*) and this was substituted with Ala. However, it should be noted that the electron density for the entire side chain of Lys113 is absent in the structures of MjGATase_SNN_109_ (PDB ID: 7D40), MjGATase_N109P (PDB ID: 7D96), and MjGATase_D110G (PDB ID: 7D95).

### MS analysis of single mutants indicates possible roles for Asp110, Tyr158, Lys151, Lys113, and Glu108 in SNN formation

**Figure 3:**
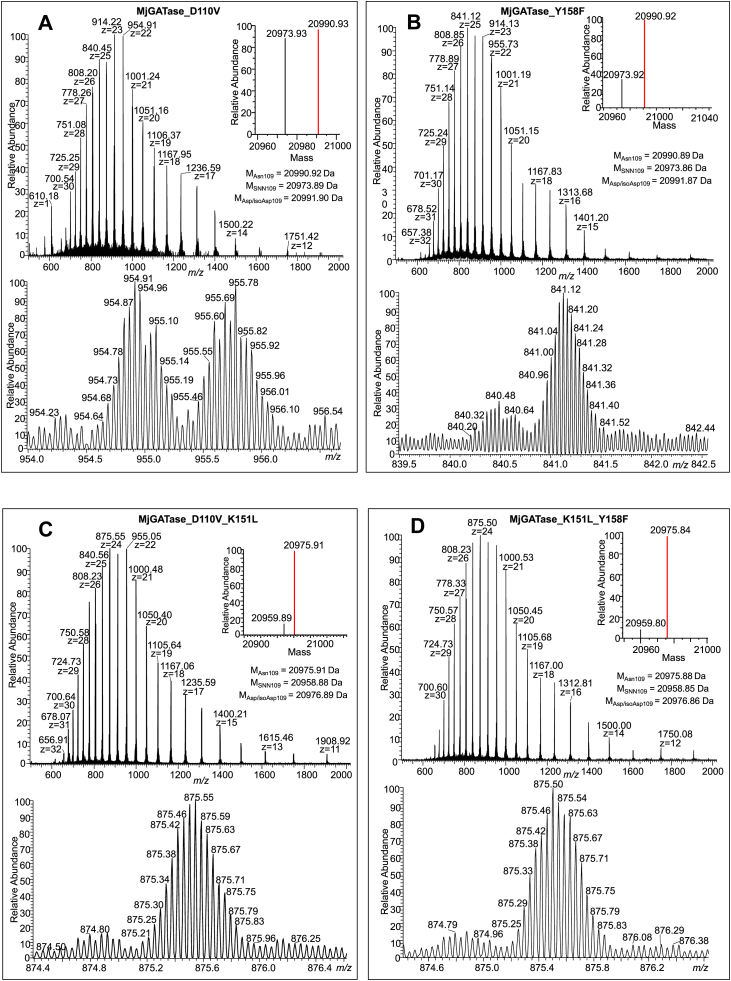
**Mass spectra of mutants of MjGATase that have retained the side chain of Asn109.** In each main panel, the *top subpanel* shows the entire mass spectrum of the protein with the inset showing the monoisotopic mass obtained after deconvolution. The *bottom panel* shows the expanded spectrum of a single charge state. The expected mass for the three different populations of the protein is provided in the *topsubpanel*. If the Asn109 containing population is identified from the spectrum, it is indicated as a *red-colored line* in the deconvoluted spectra. *A*, LC-MS of MjGATase_D110V (M_calc_ 20990.92 Da). Deconvoluted spectrum in the inset shows equal populations of the mutant with M_obs_ value of 20973.93 Da corresponding to that with SNN109 and M_obs_ of 20990.93 Da corresponding to the expected mass with Asn109. *B*, LC-MS of MjGATase_Y158F (M_calc_ 20990.89 Da). Deconvoluted spectrum in the inset shows a major population having a M_obs_ value of 20990.92 Da corresponding to the expected mass with Asn109 and a small population of 20973.92 Da corresponding to that with SNN109. *C*, LC-MS of MjGATase_D110V_K151L (M_calc_ 20975.91 Da). Deconvoluted spectrum in the inset shows a major population with M_obs_ value of 20975.91 Da corresponding to the expected mass with Asn109 and an unassigned minor population with M_obs_ of 20959.89 Da which is 16 Da lower than the expected mass of the protein. *D*, LC-MS of MjGATase_K151L_Y158F (M_calc_ 20975.88 Da). Deconvoluted spectrum in the inset shows a major population having M_obs_ value of 20975.84 Da corresponding to the expected mass with Asn109 and an unassigned minor population having M_obs_ value of 20959.80 Da which is 16 Da lower than the expected mass of the protein. Asn, asparaginyl; MjGATase, glutamine amidotransferase from *Methanocaldococcus jannaschii*; SNN, succinimide.

The single mutants of MjGATase (E108L, E108Q, K107L, E137L, K151L, K113A, Y158F, D110N, D110P, and D110V) were generated and the expression plasmids harboring the mutations were confirmed by DNA sequencing. The MjGATase_SNN_109_ and mutant proteins were purified to homogeneity and subjected to intact protein mass analysis. Figures 3, S1, and S2*A* show the intact protein mass spectra of MjGATase_SNN_109_ and all mutant proteins. As MjGATase_SNN_109_ has a stable SNN at position 109, we expected the following protein populations to be seen in the mass spectra of the MjGATase_SNN_109_ and its mutants: *viz.*, a population with SNN having a mass that is 17 Da lower than the mass expected from the sequence (M-17), a population with intact Asn having the expected mass (M), and an SNN-hydrolyzed species with a mass 1 Da higher than expected mass (M+1).

Table S1 summarizes the results obtained from intact protein mass spectrometric analysis of MjGATase_SNN_109_ and mutant proteins. The various species of MjGATase mutants observed included those having SNN, intact Asn, SNN hydrolyzed to Asp/isoAsp, and methionine oxidized forms. The relative abundance of these species was obtained from the MS data with an abundance cutoff set between 10 and 15% above the noise level. As expected, MjGATase_SNN_109_ showed a major population corresponding to the presence of SNN (Fig. S1*A*) while MjGATase_E108L, MjGATase_E137L, MjGATase_K107L, and MjGATase_K151L (Fig. S1, *B*–*E*) also exhibited a predominant SNN-containing population. Although minor, MjGATase_K151L retained a population of protein with intact Asn. MjGATase_K113A (Fig. S1*G*) also showed a minor population with intact Asn, with the reminder corresponding to protein containing SNN. With a predominant SNN population, MjGATase_E108Q (Fig. S1*H*) also behaved similar to MjGATase_E108L but retained a small population of intact Asn. MjGATase_D110V showed two populations in nearly equal proportions, corresponding to Asn109 intact and SNN (Fig. 3*A*). A dramatic increase in intact Asn population was seen in MjGATase_Y158F (Fig. 3*B*), where the SNN population was 31.7% lower in relative abundance than the noncyclized Asn109 containing protein (Table S1). MjGATase_D110N showed a major population corresponding to Asp/isoAsp (Fig. S1*F*). As expected, electrospray ionization (ESI)-MS of MjGATase_D110P, which contains an imino acid as the n+1 residue, showed complete retention of only the intact Asn109 population (Fig. S2*A*). To summarize, the intact Asn109 containing population was the highest in Y158F, followed by D110V, which had equal amounts of Asn109 and SNN, and finally small proportions of Asn109 in K151L, K113A, and E108Q mutants of MjGATase, hinting at the role of these residues in SNN formation.

### Confirmation of intact Asn presence by MS/MS analysis of peptides

**Figure 4:**
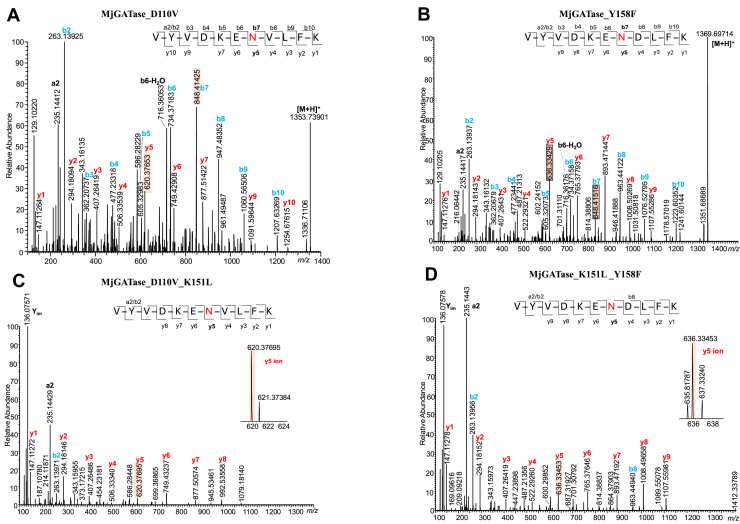
**HCD-MS/MS analysis of in-gel trypsin digested peptidescontaining residue 109showintactAsn109in specific mutants.***A*, MS/MS of peptide of *m/z* 1353.73 derived from tryptic digest of MjGATase_D110V shows the presence of intact Asn109. The y5 and b7 ions with *m/z* values of 620.37 and 848.41, respectively, are highlighted confirming Asn109. The y4 and b8 ions with *m/z* values of 506.33 and 947.48, respectively, also confirm the mutation of Asp to Val at residue 110. *B*, MS/MS of peptide of *m/z* 1369.69 derived from tryptic digest of MjGATase_Y158F shows the presence of intact Asn109. The y5 and b7 ions with *m/z* values of 636.33 and 848.41, respectively, are highlighted confirming Asn109. *C*, MS/MS of triply charged peptide of *m/z* 451.918 derived from tryptic digest of MjGATase_D110V_K151L shows the presence of intact Asn109. The y5 ion of Asn109 with *m/z* 620.37 is highlighted and the inset shows the isotope distribution of this ion. The y4 ion with *m/z* 506.33 also confirms the mutation of Asp to Val at residue 110. *D*, MS/MS of doubly charged peptide of *m/z* 685.35 derived from tryptic digest of MjGATase_K151L_Y158F shows the presence of intact Asn109. The y5 ion of Asn109 with *m/z* 636.33 is highlighted and the inset shows the isotope distribution of the y5 ion. MS/MS spectra of corresponding peptides with SNN109 or Asp/isoAsp109 are provided in Fig. S4, *A*–*D* for MjGATase_D110V and MjGATase_Y158F and Fig. S6 for the two double mutants. Asn, asparaginyl; HCD, higher-energy collision-induced dissociation; MjGATase, glutamine amidotransferase from *Methanocaldococcus jannaschii*; SNN, succinimide.

Due to the limited reliability in precisely differentiating a mass difference of 1 Da between intact Asn (M Da) and hydrolyzed SNN (M + 1 Da), the assigned Asn-intact population observed in the MjGATase mutants K151L, K113A, Y158F, D110V, and D110N could be due, at least partially, to Asp/iso-Asp population or *vice versa*. Therefore, to determine the true Asn109 intact population, in-gel trypsin digestion followed by MS/MS analysis of the derived peptides was carried out. The data are summarized in Table S2. In all the five mutants, the VYVDKEN_109_D/V/N_110_LFK/A_113_NVPR peptide was found to exist as Asn109, SNN109, or Asp/isoAsp109, albeit in varying proportions (Figs. 4, *A* and *B*, S3, *A*–*C*, S4, *A*–*F*, and S5, *A*–*D*). The relative quantification of the different populations of a peptide was made using the number of peptide spectrum matches (PSMs) (51, 52), and peptides with at least 2 PSMs and a maximum of two missed cleavages were included in the analysis. In MjGATase_K151L, the 109th residue with intact Asn was low at 5%, whereas in K113A the percentage of intact Asn109 increased to 13% (Table S2). The MjGATase_D110N also had an intact Asn109 population of 19% (Table S2). In D110V and Y158F mutants, this proportion further increased to 27 and 45%, respectively (Table S2). In comparison, in MjGATase_SNN_109_, the percentage of intact Asn109 was below 5% (Table S2, Figs. S3*D*, and S5, *E* and *F*). As expected, in the D110P mutant, all peptides containing the 109th residue had only Asn (Fig. S2*B*). In summary, substitution of the residues Asp110 and Tyr158 positively impacts the levels of the intact Asn109 population as observed in the D110V and Y158F mutants. Examination of the MjGATase_SNN_109_ structure suggests the possibility of a proton relay between side chains of Tyr158 and Lys151 (Fig. 2*B*). Based on this observation, two double mutants, D110V_K151L and K151L_Y158F were generated to examine the levels of intact Asn109 population.

### MS and MS/MS analyses of double mutants of MjGATase show high levels of intact Asn109

The intact protein mass spectra of the D110V_K151L and K151L_Y158F mutants of MjGATase showed a high proportion of intact Asn109 (Fig. 3, *C* and *D*, and Table S1). To further confirm that the observed mass corresponds to intact Asn109 and not Asp/isoAsp109, in-gel trypsin digestion followed by MS/MS analysis of the resulting peptides was carried out. Although SNN109 or Asp/isoAsp109 forms of the peptide VYVDKEN_109_D/V_110_LFK were detected (Fig. S6), their total amounts corresponded to 12.6% for D110V_K151L mutant and 18% for K151L_Y158F mutant with the Asn109 constituting the major population (Table S2). In MjGATase_D110V_K151L, the Asn at position 109 accounted for 87.4%, whereas in the K151L_Y158F mutant, the percentage was 82% (Table S2). The MS/MS analysis reassured that, in the double mutants with Asn109 intact (Fig. 4, *C* and *D*), deamidation, and SNN formation had not occurred to a significant extent. In addition, the agreement seen between intact protein MS analysis and peptide MS/MS analysis added further confidence to these findings. It should be noted that, because the masses of the two double mutants are identical, the expected mutations were confirmed by MS/MS analysis of in-gel tryptic peptides (Figs. 4*C* and S7).

### Crystal structures of D110P, D110V_K151L, and K151L_Y158F mutants of MjGATase confirm the presence of intact Asn109

As mass spectrometric data for MjGATase_D110P, MjGATase_D110V_K151L, and MjGATase_K151L_Y158F showed a complete or largely homogenous population with intact Asn109, these variants were chosen for structure determination by X-ray crystallography. The crystals of MjGATase_D110P, MjGATase_D110V_K151L, and MjGATase_K151L_Y158F diffracted to 2.4, 2.5, and 2.4 Å resolution and belonged to space groups P 3_1_ 2 1, P 4_1_, and P 4_1_, respectively, and the structures were determined by molecular replacement using MjGATase_SNN_109_ structure (PDB ID: 7D40) as the model. The data collection and refinement statistics are provided in Table 1.

**Table 1:** Summary of data collection and refinement statistics

Property	MjGATase_D110P	MjGATase_D110V_K151L	MjGATase_K151L_Y158F
PDB ID	7YC6	8GR1	8GR3
Data collection			
Wavelength (Å)	1.54179	1.54179	1.54179
Resolution range (Å)	54.63–2.4	58.00–2.5	57.73–2.4
Space group	P 3_1_ 2 1	P 4_1_	P 4_1_
Unit cell parameters			
a, b, c	63.08 Å, 63.08 Å, 101.94 Å	93.78 Å, 93.78 Å, 119.09 Å	93.41 Å, 93.41 Å, 118.25 Å
α, β, γ	90.00°, 90.00°, 120.00°	90.00°, 90.00°, 90.00°	90.00°, 90.00°, 90.00°
Total reflections	4011 (18,002)	6301 (27,987)	11,057 (49,041)
Unique reflections	365 (1391)	1159 (5174)	1304 (5761)
Multiplicity	11.0 (12.9)	5.4 (5.4)	8.5 (8.5)
Data completeness (%)	99.9 (100)	99 (100)	99.8 (100)
Mean I/σ(I)	29.6 (7.7)	12.5 (3.3)	18.6 (4.6)
Wilson β-factor (Å^2^)	16.7	10.6	6.6
R_merge_	0.06 (0.366)	0.109 (0.5)	0.089 (0.489)
R_meas_	0.062 (0.381)	0.121 (0.555)	0.095 (0.521)
CC_1/2_	0.998 (0.891)	0.989 (0.532)	0.998 (0.619)
Number of molecules per asymmetric unit	1	4	4
Refinement statistics			
R_work_	0.199	0.209	0.207
R_free_	0.221	0.247	0.241
Number of atoms			
Protein	1386	5768	5759
Ligand/ion	6	35	30
Water	76	263	285
RMSD (bond length Å)	0.011	0.0105	0.0119
RMSD (bond angle°)	1.5945	1.6283	1.6153
Ramachandran favored (%)	95	95.8	96.8
Ramachandran allowed (%)	4.5	3.8	3.1
Ramachandran outliers (%)	0.5	0.4	0.1
Clashscore	9	6	7
Average B, all atoms (Å^2^)	17	11	7

The values in parentheses are for the outer shell.

MjGATase, glutamine amidotransferase from *Methanocaldococcus jannaschii.*

**Figure 5:**
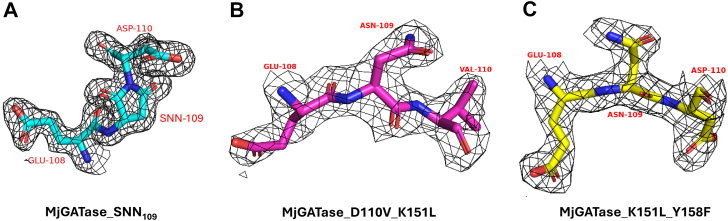
**Structural evidence for the presence of intact Asn109 side chain in the mutants.** The 2F_o_-F_c_ electron density map contoured to 1σ (*black mesh*) for residues 108, 109, and 110 in the structures of (*A*) MjGATase_SNN_109_, (*B*) MjGATase_D110V_K151L, and (*C*) MjGATase_K151L_Y158F. The electron density is evident for Asn109 in the structures of the mutants. SNN, succinimide; MjGATase, glutamine amidotransferase from *Methanocaldococcus jannaschii*; Asn, asparaginyl.

**Figure 6:**
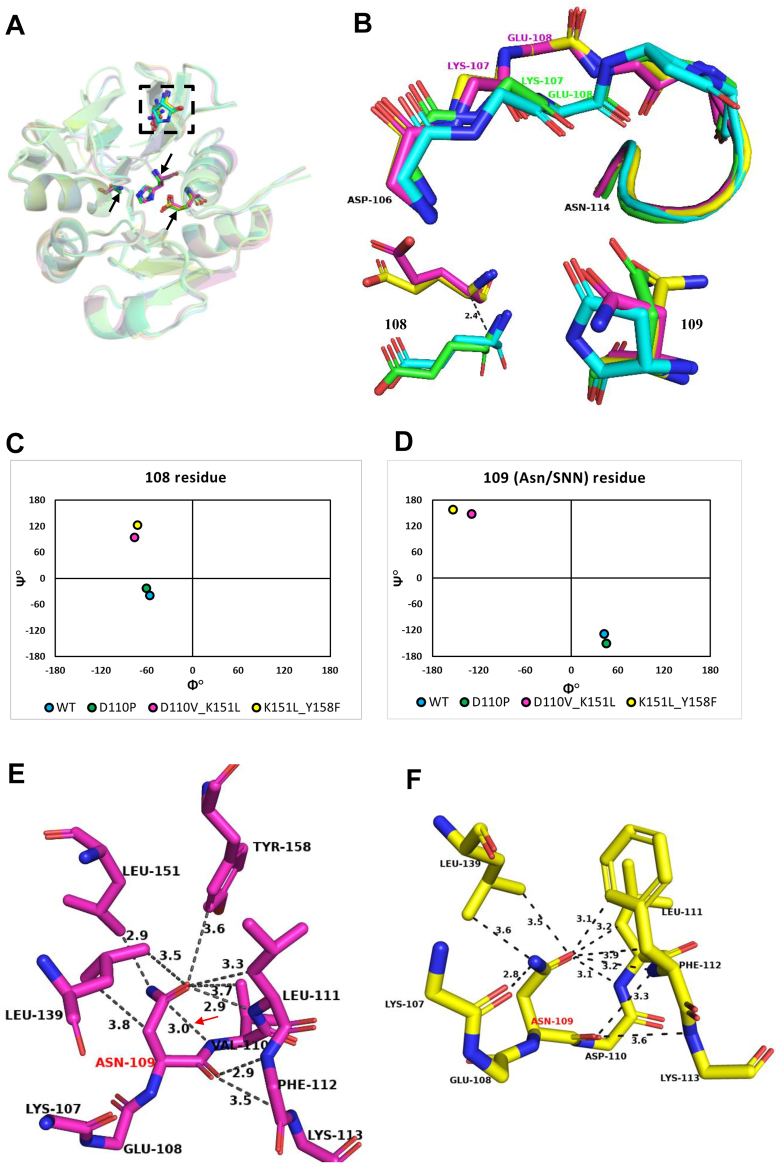
**Structural analysis of MjGATase_SNN_109_ and mutants.***A*, superposition of the structures of the mutants D110P (*green*), D110V_K151L (*purple*), and K151L_Y158F (*yellow*) on the MjGATase_SNN_109_ (*cyan*) did not show dramatic differences in the overall structure. SNN/Asn at residue 109 is shown in *stickrepresentation* and *boxed*. The active site of the enzyme comprising of Cys76, His163, and Glu165 is shown in *stickrepresentation* and indicated by *arrows*. *B*, the *top panel* shows the superposition of the loop harbouring Asn109/SNN109 from residues 106 to 114. The backbone of residues from 106 to 110 is shown in *stick* and residues 111 to 114 are shown in *cartoon representation*. The *bottom panels* show the zoomed in view of the superposition of the residues 108 and 109 along with their side chains. The nonsuperimposability of the CA of Glu108 in the MjGATase_SNN_109_ on to their counterpart in the double mutants shows the lateral movement of this segment in the structures of the mutants. The *dashed line* indicates the movement of Glu108 by 2.4 Å. Note that Glu108 in the structure of MjGATase_D110P overlays well on to Glu108 in the MjGATase_SNN_109_ structure. Colors used are as indicated in *panel* (*A*). *C*, Ramachandran map occupancy of the residue 108 in WT and mutants. *D*, Ramachandran map occupancy of residue 109 in WT and mutants. WT in (*C* and *D*) refers to MjGATase_SNN_109_ structure. Contacts of Asn109 at 4 Å distance cutoff in the structures of (*E*) MjGATase_D110V_K151L and (*F*) MjGATase_K151L_Y158F. The numbers adjacent to the dashed lines represent distance in Å**.**The *arrow* in (*E*) points to the near-attack conformation distance between the backbone amide of Val110 and CG of Asn109. Asn, asparaginyl; MjGATase, glutamine amidotransferase from *Methanocaldococcus jannaschii*; SNN, succinimide.

In contrast to MjGATase_SNN_109_, in the structures of the three mutants, the electron density for Asn109 was clearly evident (Figs. 5 and S2*C*), corroborating the findings from MS. The overall structures of the mutants superpose well with the MjGATase_SNN_109_ structure with RMSDs of 0.412 Å for MjGATase_D110P, 0.3 Å for MjGATase_D110V_K151L, and 0.24 Å for MjGATase_K151L_Y158F. The superposition of all the three mutant structures on the WT is shown in Figure 6*A*.

### Structure analysis of the mutants

The loop (106–114 residues) harboring Asn109 in MjGATase_D110P superposes well with that in the MjGATase_SNN_109_ structure. However, in the structure of the double mutants, there is a variation in the backbone conformation from residues Lys107 to Leu111 (Fig. 6*B*). This shift is clearly evident in the dihedral angles ϕ and ψ of residues Glu108 and Asn109 in the structures of the double mutants MjGATase_D110V_K151L and MjGATase_K151L_Y158F, where they occupy different quadrants of the Ramachandran map compared to that in the MjGATase_SNN_109_ structure (Fig. 6, *C* and *D*). In the WT structure, the dihedral angles ϕ (42.6°) and ψ (−128.8°) of SNN109 fall in the bottom-right quadrant of the Ramachandran map. The MjGATase_D110P structure has the Asn residue adopting ϕ and ψ values of 45.1° and −150.5°, respectively, also lying in the bottom-right quadrant, as seen for the WT enzyme. In contrast, in MjGATase_D110V_K151L and MjGATase_K151L_Y158F structures, the dihedral angles of the corresponding Asn109 fall in the top-left quadrant of the Ramachandran map, with values of ϕ = −129.3°, ψ = 148.6°, and ϕ = −153.6°, ψ = 157.8°, respectively (Fig. 6*D*).

The SNN-containing loop in the MjGATase_SNN_109_ has an α-turn stabilized by a hydrogen bond between Glu108 C=O and Phe112 NH and a β-turn with a hydrogen bond between Glu108 C=O and Leu111 NH (Fig. S8*A*). These features are retained in the D110P mutant (Fig. S2*D*). This α-turn and β-turn initiating from Glu108 in the WT are replaced by an α-turn and β-turn initiating from Asn109 in D110V_K151L and K151L_Y158F MjGATase mutants. The α-turn involves Asn109, Val110/Asp110, Leu111, Phe112, and Lys113 with hydrogen bonding interaction between Asn109 C=O and Lys113 NH, and β-turn involving Asn109, Val110/Asp110, Leu111, and Phe112 with hydrogen bonding interaction between Asn109 C=O and Phe112 NH (Fig. S8, *B* and *C*). The α-turn in the MjGATase_SNN_109_ is facilitated as Glu108 backbone C=O points inward into the loop with ϕ = −56.3°, ψ = −38.7°, whereas in the double mutants, a change in the dihedral angle ψ of Glu108 to 94.2° and 122.5° in MjGATase_D110V_K151L and MjGATase_K151L_Y158F, respectively, leads to a reorientation of backbone C=O (Figs. 6*C* and S8). The conformation adopted by Asn109 being dissimilar from that of the SNN109, alters the backbone orientation of the preceding residue, Glu108, in the double mutants (Fig. 6*B*).

In the three structures where Asn109 is intact, the Asn109 side chain adopts three different conformations (Fig. 6*B*, bottom right panel). The side chain dihedral angles χ1 and χ2 of Asn109 are 72.9° and 130.8°, 173.9° and −99.6°, and 58.2° and 93.1° in MjGATase_D110P, MjGATase_D110V_K151L, and MjGATase_K151L_Y158F, respectively. The corresponding dihedral angles χ1 and χ2 in the WT harboring SNN are 127.3° (χ1) and 179.4° (χ2). Structural superposition of the loop harboring SNN/Asn in WT and the mutant structures showed that the Asn109 conformation in K151L_Y158F MjGATase is drastically different from that of SNN, whereas, in the D110V_K151L MjGATase structure, it closely resembles the SNN conformation, mimicking the “near-attack” conformation (χ1 of +120° and χ2 of +90° or −90°, (3)) (Fig. 6*B*, bottom right panel). The distance proposed between the backbone amide NH of the n+1 residue and CG of Asn (n^th^ residue) for the “near-attack” conformation favoring cyclization is < 3.5 Å (3, 37, 53, 54) and the corresponding distance in the structure of MjGATase D110V_K151L is 3.0 Å (Fig. 6*E*).

In the MjGATase_SNN_109_ structure, most contacts involving SNN arise from the OD1 atom (Fig. 2*C*). Similarly, in the double mutant structures, most interactions of the Asn109 side chain are with the OD1 atom (Fig. 6, *E* and *F*). In both the double mutant structures, OD1 of Asn109 makes an H-bonding interaction with the backbone NH of Leu111 (Fig. 6, *E* and *F*). This contact is retained after SNN formation, as observed in the MjGATase_SNN_109_ structure, and is also retained in the D110P structure, where Asn109 cannot form SNN (Figs. 2*C* and S2*E*). In MjGATase K151L_Y158F, the OD1 atom of Asn109 makes an additional H-bonding interaction with backbone NH of Phe112 and ND2 makes H-bonding interaction with backbone C=O of Lys107 residue (Fig. 6*F*). These additional H-bonds of Asn109 in the K151L_Y158F structure may explain why the Asn109 conformation differs from that in the D110V_K151L structure. The other interactions of the OD1 atom in both the double mutant structures are van der Waals in nature.

The acidity of backbone amide hydrogen of the n+1 residue is mainly dictated by the backbone dihedral ψ adopted by the n+1 residue (37, 55). The dihedral angles adopted by Val110 are ϕ = −46.3° and ψ = −34.1° in the structure of MjGATase_D110V_K151L and ϕ = −59.3° and ψ = −32.8° for residue Asp110 in the structure of MjGATase_K151L_Y158F. The corresponding backbone dihedrals for Asp110 are ϕ = −100.2° and ψ = −47.5° in the MjGATase_SNN_109_ structure. In the CMD simulation of MjGATase_ASN_109_ (discussed below), the ψ value of Asp110 ranged from −10° to −50° for Asp110 (Fig. S9*A*). In the plot of relative proton affinity (2-acetamido-N-methylacetamide and N-formyl-glycinamide) as a function of dihedral angles (37, 55), these conformations lie in the moderately low proton affinity regions, hence, the backbone amide hydrogen of the n+1 residue is moderately acidic in MjGATase.

### Mutants with intact Asn109 exhibit lowered thermal stability without variation in activity

**Figure 7:**
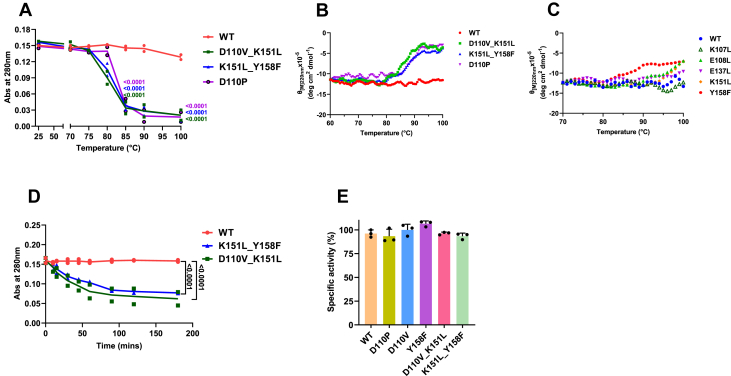
**Modification of Asn109 to an SNN is required for the hyperthermostability of MjGATase but not for the enzymatic activity.***A*, thermal melting curve of the mutants D110P, D110V_K151L, and K151L_Y158F and WT of MjGATase obtained using UV absorbance. The graph shows the data points of two independent measurements with the line connecting the means. Abs along *y*-axis denotes absorbance. The *p* values reported at 85 °C and 100 °C arises from two-way ANOVA with Dunnett’s test compared with that of WT. *B* and *C*, thermal melting curve of WT and mutants obtained using CD. *D*, the rate of unfolding at 80 °C plotted for the double mutants and WT. The scatterplot shows values from two independent measurements with the line connecting the means. The *p* value from two-way ANOVA analysis with Dunnett’s test is shown for the last data point. *E*, specific activity normalized to WT enzyme activity. Values of mean are plotted with error bars representing SD from three replicates. Asn, asparaginyl; MjGATase, glutamine amidotransferase from *Methanocaldococcus jannaschii*; SNN, succinimide.

The thermal stability of MjGATase_D110P, MjGATase_D110V_K151L, and MjGATase_K151L_Y158F was examined using UV absorbance of soluble fraction obtained post sample heating, as well as by far-UV CD. The absence of the SNN modification resulted in lower melting temperatures (Fig. 7, *A* and *B*) for these mutants, with T_m_ values ranging between 82 and 84 °C (as deduced from Fig. 7*A*), whereas the WT did not unfold even at 100 °C (38). The rate of unfolding at 80 °C was also measured for the double mutants and the WT protein. Both double mutants, within a few minutes showed rapid unfolding with a sharp decline in the slope of the unfolding curve, whereas the WT protein did not unfold even after 180 min (Fig. 7*D*). In contrast, the thermal melting curves of mutants retaining SNN_109_ did not differ from that of the WT (Fig. 7*C*), with the exception of Y158F, where some degree of protein unfolding was observed. This behavior may be attributed to the presence of ∼45% Asn109-containing population in this mutant. REST2 MD simulations on mutants where Asn109 was replaced by Ser and Pro showed at higher temperatures, structural destabilization, of the distal β-sheet originating from the SNN loop (40). Taken together, the current study also implicates a critical role for the SNN modification in maintaining hyperthermostability. The mutants, D110V, Y158F, D110P, D110V_K151L, and K151L_Y158F of MjGATase that retain Asn109 exhibited no change in enzymatic activity compared to the WT (Fig. 7*E*), indicating that SNN does not play a role in catalysis. This has been earlier observed in MjGATase N109S, D110G, and D110K that lack SNN modification (38).

### Formation of SNN at low pH in MjGATase_D110V_K151L

To investigate whether the intact Asn109 population of MjGATase_D110V_K151L can convert to SNN at lower pH, the WT and the mutant were incubated at pH 3 for 16.5 h at 60 °C and subsequently examined using MS. The mass spectrum of MjGATase_D110V_K151L showed an increase in the SNN109 population from 17 to 34% over a period of 16.5 h relative to the Asn109-containing population (Fig. S10, *A* and *B*). The presence of the SNN population demonstrates the ability of the mutant to form SNN at low pH, albeit at a very slow rate. This detection of SNN despite the absence of the putative catalytic base (Asp110) in the mutant suggests that the overall active site geometry is optimized for very high deamidation rates. It should be noted that under crystallization conditions (pH 6.5, 25 °C and incubation for 10 days prior to data collection), the Asn109 side chain remains intact, as supported by the observed electron density.

### QM/MM metadynamics

#### *In silico*generation of MjGATase_ASN_109_ for QM/MM studies

**Figure 8:**
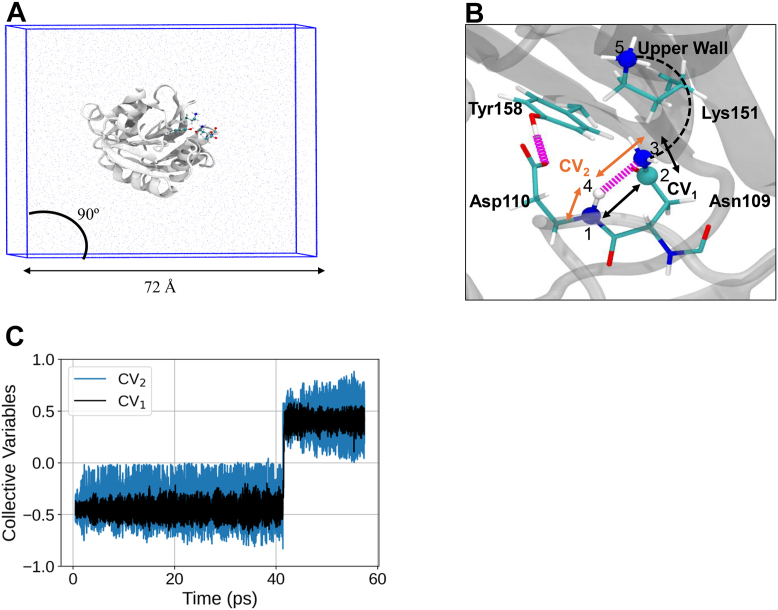
**System under computational study, initial conformation of the QM region, and time evolution of the collective variables.***A*, MjGATase_ASN_109_ is shown in *new cartoon representation* in *white*. QM region consisting of residues Asn109, Asp110, Lys151, Tyr158, and backbone carbonyl of Glu108 is highlighted in Corey-Pauling-Koltun (CPK) representation. Water molecules are shown as *iceblue dots*. Ions are not shown for clarity. *B*, zoomed view of the QM region used for QM/MM MD metadynamics simulation. Atoms within QM region are shown in *Licorice representation*. The remaining protein structure is partly shown in *white transparent mode* in *new cartoon representation.* Water molecules and ions are not shown for clarity. Hydrogen bonds are shown as *magenta springs*. Atoms involved in the CV definitions and upper wall definition are highlighted in CPK representation. Atoms 1, 2, and 3 are part of CV_1_ (*black arrows*) and atoms 1, 3, and 4 are part of CV_2_ (*orange arrows*). The upper wall (*black dashed line*) is between atoms 3 and 5. *C*, time evolution of the two collective variables. CV__1__ and CV__2__ show a single transition at around approximately 40.75 to 41.05 ps. Asn, asparaginyl; MD, molecular dynamics; MjGATase, glutamine amidotransferase from *Methanocaldococcus jannaschii*; QM/MM, quantum mechanics/molecular mechanics; SNN, succinimide.

To gain further insights through QM/MM studies, into role of neighboring residues on SNN formation, using Gaussview (56) we first generated a modelled structure of MjGATase wherein the Asn109 side chain is intact. This was followed by energy minimization and equilibration at constant temperature, pressure, and volume using GROMOS force field (40) and thereafter, AMBER99sb-star-ILDN (57, 58) (https://modelarchive.org/doi/10.5452/ma-vn1er). This structure from CMD, referred to as MjGATase_ASN_109_ (Fig. 8*A*), was used for subsequent QM/MM MD metadynamics simulations, with and without the Gaussian bias. Interestingly, the overall structural superposition of MjGATase_ASN_109_ on MjGATase_SNN_109_, and the double mutants yielded similar RMSD values (0.716 Å, 0.753 Å, and 0.739 Å, respectively, for D110V_K151L, K151L_Y158F, and MjGATase_SNN_109_), a feature also seen in the loop containing Asn/SNN and in the extended β-sheet region (Fig. S11). Structural overlay of MjGATase_ASN_109_ on MjGATase_D110V_K151L showed similar disposition of the side chain of Asn109 in the near attack conformation (3, 37, 53, 54) with a van der Waals distance between CG of Asn109 and Asp110 backbone NH (Fig. S11*G*). Examination of the CMD simulation data for the possibility of any of 10 Asn side chains adopting a near attack conformation showed that only Asn109 favored this conformation with a mean distance of 3 Å (Fig. S9*B*).

### Results from QM/MM metadynamics

#### Movement of the reactant to the product in the dihedral angle space

In the QM/MM MD metadynamics simulations, the reaction was represented through two collective variables, one describing the cyclization and deamidation and the other describing the deprotonation and assistance for deamidation. The run length was ∼57 ps starting from the structure as shown in Figure 8*B* with atoms of Asn109, Asp110, Lys151, Tyr158, and backbone C=O of Glu108 considered in the QM region and the rest of the protein in the MM region. The trajectory yielded only 1 transition from the reactant basin to the product basin as shown in Figure 8*C*.

Some of the Ramachandran angles and side chain dihedral angles from the quantum region were compared with those of MjGATase_ASN_109_ and MjGATase_SNN_109_ (Fig. S12, *A–F*). In the Ramachandran plots, while simulations show product state in the bottom-left quadrant (Fig. S12*C*), the experimental product state is present in the bottom-right quadrant (Fig. S12*B*). However, SNN-containing PDB structures display points in both the bottom quadrants of the Ramachandran plot (Fig. S12*G*), indicating that ϕ can occur in both the bottom quadrants with a restriction of ψ between −105° and −145°, coming from Asn cyclization (Fig. S12*G*). In the case of side chain dihedrals of Asn109, Janin plots (Fig. S12, *D–F*, (59)), suggested the partial sampling of the SNN state (Fig. S12*E*) in the QM/MM metadynamics trajectory reaction run (Fig. S12*F*).

### Asp110 sidechain abstracts its own backbone proton

**Figure 9:**
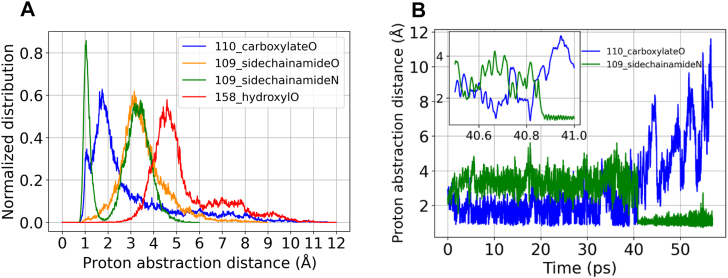
**Analysis of the QM/MM MD metadynamics simulation.***A*, normalized distance distributions for the Asp110 backbone amide proton abstraction by the indicated neighboring nucleophilic sites. *B*, time evolution of the Asp110 (n+1) backbone amide proton abstraction by its own side chain carboxylate and by the side chain of Asn109 through the nitrogen atom. Inset shows a zoomed view at time around 40 ps. Asn, asparaginyl; MD, molecular dynamics; QM/MM, quantum mechanics/molecular mechanics.

To explore the possibility of the (n+1) residue backbone amide hydrogen to bond with any neighbouring basic sites, we calculated the normalized distributions of the corresponding proton abstraction distances. The mutational data presented in the preceding section clearly establish the key role of the proximal carboxylate group of Asp110 in determining the extent of SNN formation and earlier studies further implicate Asp110 in impeding aqueous hydrolysis through a combination of steric and electronic effects (38, 40). The carboxylate group with a pKa of 3.4 ± 1 (60, 61) often functions as a base in enzymatic reactions. Figure 9*A* summarizes the computed distribution of the proton abstraction distance of the Asp110 carboxylate and also presents comparative plots for other proximal groups Tyr158 hydroxyl O, and the Asn109 side chain amide O and N atoms. Asp110 CG oxygen shows a probability of 0.64 at an abstraction distance of < 2 Å, demonstrating Asp110 is abstracting the hydrogen (a distance of 1 Å), while failing to retain it (Fig. 9*B*). The possible scenarios are: the proton is transferred back to Asp110 amide NH or to a water molecule. Although water-assisted deamidation mechanisms are studied in synthetic peptides (53, 54, 62), we have not explored its role in SNN formation in MjGATase. Interestingly, the highest probability (0.86) at a proton abstraction distance of 1 Å is that of Asn109 amide NG (Fig. 9*A*), which until 40 ps did not abstract the Asp110 backbone proton (Fig. 9*B*). However, at around 40 ps, the side chain possibly assumes the near attack conformation, by which it directly abstracts the proton and retains it as seen up to 57 ps (Fig. 9*B*). The deviation of the distance to Asp110 CG oxygen from 1 Å coincides with the proton abstraction by Asn109 amide NG, without exchange between them. Of the two, the more frequent event is Asp110 carboxylate abstracting the proton.

Significant contacts in the MD simulation runs that are present in the crystal structures of either MjGATase_SNN_109_ or mutants are described below. The interaction between OD1 of Asn109 and backbone amide hydrogen of Leu111 was found to be retained (population: 40–45%) in the QM/MM MD and QM/MM MD metadynamics runs (Fig. S13, *A* and *B*), while the backbone oxygen of Asn109 and backbone amide hydrogen of Phe112 were found to be retained at a much lower extent of 12% and 25% in the QM/MM MD and QM/MM MD metadynamics runs, respectively (Fig. S13, *C* and *D*). The contact between the backbone oxygen of Asp110 residue and backbone amide H of Lys113 residue was retained in all three simulations to only about 30% (Fig. S13, *E* and *F*).

From the deposited hills in the collective variables (CV) space (Fig. S14*A*), we extracted a minimum free energy path starting from the reactant state point to an arbitrary point on the landscape. The final point is not the product point, as we were unable to fill the product basin significantly, due to short length of the QM/MM MD metadynamics trajectory. Hence, we proceeded to estimate the activation barrier from the reactant basin. In Fig. S14*A*, the minimum free energy path joining the reactant state (represented by coordinate [−0.4, −0.4] in the CV space) to a point 3 (represented by coordinate (0.0, +0.4) in the CV space) is shown as black dashed line and highlights four state points that is reactant, 1, 2, and 3. Point 3 lies *en route* to the product basin (state point 4) and 1 is the barrier between reactant and state point 2. The free energy profile for the segment between states 1 and 2 is shown separately in Fig. S14*B*, where the *x*-axis corresponds to the number of nodes used in the minimum path-finding algorithm. It provides an activation barrier of ∼3.4 kcal/mol for the deprotonation of the n+1 backbone amide.

### States on the CV space and their structures

**Figure 10:**
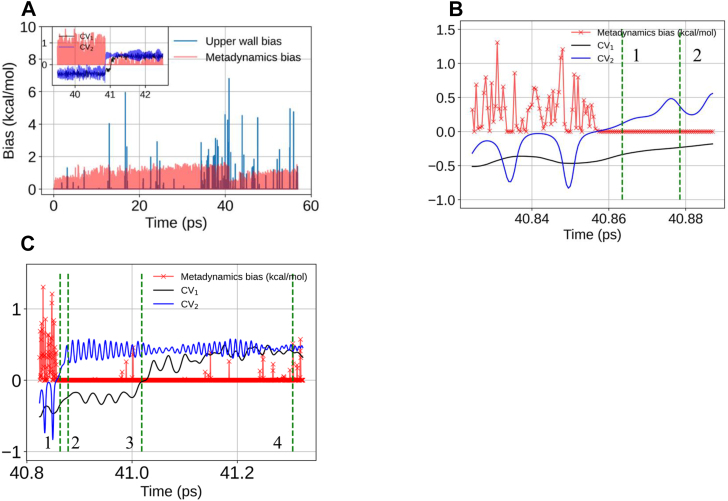
**Time evolution of biases and collective variables.***A*, time evolution of the metadynamics bias and upper wall bias. Inset shows a zoomed view of the time evolution of the two collective variables along with the metadynamics bias. *B*, a further zoomed view of panel (*A*) near the time point where metadynamics bias has filled up the reactant basin. State 1 and 2 from Fig. S14 (*A*) are highlighted with *dotted vertical green lines*. *C*, time advancement toward the product basin. States 1 to 4 from Fig. S14 (*A*) are highlighted with *dotted vertical green lines*.

The states on the CV space were monitored using the metadynamics bias and the two CVs are shown in Figure 10. In addition, we had an upper wall bias potential. The reactant basin is filled by the metadynamics bias at around 41 ps (Fig. 10*A*). The inset shows the metadynamics bias along with the two CVs. Figure 10*B* shows the same for a stretch of time points, where the metadynamics bias is 0 and CV_2_ crosses 0 value in the *y*-axis, while the CV_1_ approaches 0. Time points corresponding to the four state points, 1 to 4 of Fig. S14*A* are shown with vertical dashed green lines in Figure 10, *B* and *C*. At state point 3, CV_1_ crosses the zero value on the *y*-axis (Fig. 10*C*). To move from state point 2 to 3 and then from 3 to 4, the system confronts only small barriers, because we observe that the additional metadynamics bias is fairly low, at a value of ∼0.5 kcal/mol. Near state point 4, both the CV values fluctuate around +0.5, corresponding to the product state.

Together, Figures 10 and S14 provide insights on the free energy surface up to state point 2. Point 1 is the first activated state from the reactant basin with a free energy barrier for the deprotonation step of 3.4 kcal/mol. Since we could not observe the product to reactant transition, we do not have a conclusive barrier height for the cyclization and deamidation steps of SNN formation.

We present the structures of the four state points (green dashed lines, Fig. 10*C*) in Fig. S14*C*. State point 1 corresponds to the structure where the backbone amide hydrogen of Asp110 is detached from the nitrogen and is near the nitrogen nucleus of the side chain amide of Asn109. State point 2 corresponds to the structure where the backbone amide H of Asp110 now belongs to the side chain amide nucleus of Asn109 completely that is C-NH_3_ unit has formed but the loss of ammonia has not happened as the nitrogen is still attached to the side chain amide carbonyl-carbon of Asn109. State point 3 corresponds to the partial cyclization and deamidation, which goes to completion at state point 4, which is the product state.

## Discussion

The spontaneous deamidation of Asn residues in peptides is primarily influenced by the succeeding residue, with faster deamidation rates observed when the following residue is glycine. In proteins, however, the rate is influenced by both the primary sequence and the three-dimensional structure (30, 32, 35, 36, 37). Although in peptides, the half-life (t_1/2_) for deamidation of an Asn residue when followed by an aspartyl residue is higher than when glycine, serine, or alanine occupy the n+1 position (2, 20), in MjGATase, the Asn109 residue followed by Asp undergoes spontaneous and complete deamidation, leading to the formation of a stable SNN (38). This indicates that the three-dimensional architecture of MjGATase plays a major role in the deamidation process, supported by the fact that Asn7, followed by a glycyl residue, does not undergo deamidation in the same protein. Our mass spectrometric and crystallography studies of MjGATase mutants, followed by QM/MM analyses, provide mechanistic insights into Asn109 deamidation. The search for mutants with masses corresponding to unmodified Asn109 suggested that the residues Tyr158, Asp110, Lys113, Lys151, and Glu108 have a role in SNN formation, in decreasing order of their involvement. Further mutational analysis showed that the double mutants D110V_K151L and K151L_Y158F of MjGATase exhibited the highest proportion of intact Asn109. Crystal structures of these double mutants confirmed the presence of intact Asn109. Although the single mutant K151L had a negligible population of intact Asn109, the double mutants D110V_K151L and K151L_Y158F exhibited a dramatic increase in intact Asn109 population. These findings indicate that the three-dimensional environment of the loop harboring SNN/Asn109 is sensitive to local conformational changes and that multiple modifications in this environment can lead to the abrogation of SNN formation. Lysine residues that are spatially proximal to an asparagine residue can increase the rate of deamidation by stabilizing the negative charge on the transition state prior to tetrahedral intermediate formation (63). This interaction may be facilitated by field or inductive effects (63). In MjGATase, Lys151 and Lys113, which are spatially close to Asn109, may play a similar role in favoring SNN formation. Studies on penta/hexapeptides with asparagine followed by a serine residue have established higher rates of deamidation (2, 20, 30, 55). In addition, it has been observed that Ser at n+1 position promotes SNN formation (32, 64, 65) possibly through the hydroxyl oxygen of the Ser residue H-bonding with the backbone amide or its hydroxyl hydrogen making an H-bond with either side chain Asn oxygen or nitrogen atoms (30). This, in turn, can increase the nucleophilicity of the backbone amide of the n+1 residue or increase the electrophilicity of the Asn side chain carbonyl carbon, thus facilitating SNN formation (30, 32, 64, 65). Similarly, in MjGATase, the side chain of Asp110 may form H-bond with its own backbone amide NH, thereby increasing its nucleophilicity. In addition, the hydroxyl group of Tyr158, could interact with the Asn side chain OD1 and ND2 atoms, increasing the electrophilicity of the CG atom. Additionally, Tyr158 could participate in proton relay after Asp110 abstracts the proton. The residue at the n+1 position (Asp/Val110) in both the double mutant crystal structures and CMD simulation of MjGATase_ASN_109_ adopts a ψ backbone dihedral angle that enhances the acidity of backbone amide hydrogen (37, 55). Hence, proton abstraction from the backbone amide following Asn109 is feasible in MjGATase. The n+2 residue can also assist the deamidation reaction, as observed in TPI (37). In TPI, a hydrogen bond between the backbone amide of the n+2 residue and the side chain OD1 of Asn71 facilitates the near-attack conformation required for SNN formation (37). A similar interaction is observed between the Asn109 side chain OD1 and the backbone amide of Leu111 (n+2) in both double mutant structures of MjGATase and this interaction is also retained after SNN formation. Hence, the n+2 residue could promote SNN formation by positioning the Asn in the right orientation.

An optimal conformation of the Asn backbone dihedral angle ψ of −120°, side chain dihedrals χ1 of +120°, χ2 of +90° or −90°, and CG_n_-N_n+1_ distance <3.5 Å enables SNN formation (3, 6, 53, 54). Among different conformations seen in MD simulations, adopted by Asn373 in the P-domain of norovirus capsid protein, a rare *syn* (ϕ = −180 °and ψ = 0°) backbone conformation of Asn373 was observed. In this conformation, eclipsing of the Asn373 nitrogen by the nitrogen of Asp374 could increase the nucleophilicity of the backbone n+1 amide, leading to deamidation (36). In MjGATase, a *syn* conformation for Asn109 was not observed in the energy-minimized structure of MjGATase_ASN_109_ obtained from CMD simulations. The Asn373 residue that undergoes deamidation in Saga P-domain of the capsid protein lies on a surface exposed loop, is preceded by a Glu residue, and is followed by an Asp residue (9). This arrangement is similar to the MjGATase sequence Glu_108_Asn_109_Asp_110_, where Asn109 also lies on a loop (38, 40). Despite these similarities, the deamidation is facilitated by different factors in these proteins primarily due to differences in three-dimensional structures.

QM/MM MD simulations with free energy methods using umbrella sampling (66, 67) and metadynamics (68, 69, 70) have enabled the elucidation of detailed mechanisms and associated free energies of enzymatic catalysis. In MjGATase, the QM/MM analysis of the reaction path, using the collective variable description, supports neighboring group participation, suggesting that proton abstraction from the Asp110 backbone NH by the side chain carboxylate of Asp110 is plausible. However, the pathway of this proton beyond its transfer to the carboxylate remains unknown. The only other functional group proximate to Asp110 NH is the side chain amide of Asn109, as seen in the CMD simulated structure. The activation barrier for deprotonation is about 3.4 kcal/mol, a low value. From QM/MM and Perturbed Matrix Method studies on peptides, with and without including water explicitly, it has been observed that the energy barrier for the overall SNN formation reaction is in the range of ∼20 to 25 kcal/mol (49, 62) or ∼30 to 40 kcal/mol (37, 48, 54, 71, 72) respectively, with the former being in agreement with the experimental estimate (6, 73). From earlier work on TPI (37), the energy barrier for the tetrahedral intermediate formation has been shown to be higher at 30 to 40 kcal/mol. In TPI, unlike MjGATase, SNN formation at Asn71 is a very slow process over days (37.8 days). In contrast, the entire population of recombinant MjGATase isolated from *Escherichia coli* is the SNN form (38), precluding an easy measurement of the rate of conversion. Therefore, we expect the rate of the autocatalytic reaction in MjGATase to be significantly higher than in TPI. Furthermore, as we have not proceeded in our QM/MM analysis beyond the deprotonation step, it is possible that a subsequent step—either formation of tetrahedral intermediate or ammonia release—could have a higher activation barrier.

In conclusion, we find an agreement between experimental observations on mutants and QM/MM MD metadynamics simulations. Mass spectrometric analysis of D110V and D110N show continued formation of SNN, indicating an alternative mechanism for the removal of the n+1 proton. The near-attack conformation for Asn109, revealed from the simulations, suggests an alternative path for abstraction of the Asp110 NH proton—a process that could be enabled by the hydroxyl group of the neighboring Tyr158 side chain and the NE amino group of the Lys151 side chain.

Our mutational analysis yields an apparently paradoxical result. The K151L single mutant forms the stable SNN efficiently unlike the Y158F and D110V single mutants that show significantly impaired SNN formation suggesting direct role in autocatalysis and subsequent stabilization of the SNN. Yet, in the double mutants, the K151L mutation appears to dramatically enhance the impairment of SNN formation in Y158F and D110V single mutants. It is possible that Lys151 plays a supporting role in SNN formation and stabilization, which offsets single mutations at positions Tyr158 or Asp110. Interestingly, inspection of a limited dataset of 84 sequences of GATase from archaea reveals an almost complete conservation of Lys/Arg151, whereas a very small number of examples containing the Y158F and Asp/Glu110G mutations were found (40). Protein sequences are shaped over evolutionary timescales with selected pressures being driven by biochemical function and structural stability. The cluster of residues Asp110, Tyr158, and Lys151 which are spatially proximal to the stable SNN in MjGATase, present an example of evolutionary selection of a spontaneous PTM that maybe an essential requirement for a protein to remain functional in an organism living in deep-sea hydrothermal vents.

Additionally, our findings on the structure-stabilizing role of SNN in MjGATase provides motivation to search for proteins containing stable SNN, which may, in turn, contribute to structure stabilization. The structure of GATase (PDB ID: 1WL8) from the archaeon *Pyrococcus horikoshii* also has an SNN at the same location, arising from the dehydration of an aspartyl residue, and this modification may impart structural stability to the protein from a thermophile.

## Experimental procedures

### Generation of mutants

The protein under study is glutamine amidotransferase (E.C. 6.3.5.2), a subunit of GMPS from *M. jannaschii* (UniProt accession number: Q58970). The primer pairs with the desired mutation, custom synthesized from Sigma-Aldrich or Eurofins, Bengaluru are listed in Table S3. The MjGATase mutants Y158F, E108Q, K113A, D110V, and D110N were generated by quick change PCR with P1/P2, P19/20, P21/P22, P3/P4, and P23/P24 primers, respectively. The pST39 plasmid with MjGATase gene (38) was used as a template for PCR amplification. DpnI digestion was carried out after PCR and *E. coli* XL10 Gold-competent cells were transformed with the digested DNA. The plasmid was isolated and sequenced to check for the presence of the mutation.

For the generation of E108L, K107L, K151L, E137L, and D110P mutants of MjGATase, overlap PCR was used to generate two amplicons with the primer pairs P15 and RP of the desired mutant, and P16 and FP of the desired mutant using WT as a template. The gel-extracted amplicons were used as templates for the generation of the third amplicon using P15/P16 primer pairs, which were also gel-extracted and digested using XbaI and KpnI. pST39 plasmid with MjGATase and MjATPPase genes was used as a vector and restriction digested using XbaI and KpnI. Ligation of the gel-extracted vector and insert with T4 DNA ligase was carried out and *E. coli* DH5α or XL10 Gold-competent cells were transformed with the ligated DNA. After screening by PCR, the plasmids from the positive clones were isolated and verified by DNA sequencing for the presence of the mutation.

For the double mutants of MjGATase, overlap PCR was used to generate two amplicons with the primer pairs P4 and P15, and P3 and P16 using MjGATase_K151L as the template for the D110V/K151L mutant and with the primer pairs P18 and P15, and P17 and P16 using MjGATase gene as a template for K151L/Y158F mutant. The gel-extracted amplicons were used as templates for the generation of the third amplicon using P15/P16 primer pairs. pST39 plasmid with MjGATase and MjATPPase genes restriction digested using XbaI and KpnI was used as a vector. The gel-extracted vector and insert were used for AQUA cloning (74) and *E. coli* DH5α-competent cells were transformed with the DNA mix. After screening by PCR, the plasmids from the positive clones were isolated and verified by DNA sequencing for the presence of the mutations.

### Protein expression and purification

The procedure described earlier (38) was adopted for protein expression and purification with minor modifications. Briefly, *E. coli* Rosetta (DE3) pLysS cells carrying either the pST39_MjGATase or mutant plasmid were grown in 800 ml of Terrific Broth with ampicillin (100 μg ml^-1^) and chloramphenicol (34 μg ml^-1^) till OD_600_ of 0.4 to 0.6, induced with 0.3 mM IPTG, and grown for another 3 h at 37 °C. Thereafter, the culture was centrifuged at 3400*g* for 10 min to pellet the cells. Cells resuspended in 20 ml of fresh lysis buffer (20 mM Tris–HCl, pH 7.4, 10% glycerol, 0.1 mM EDTA, 2 mM DTT, and 0.1 mM PMSF) and lysed using a probe sonicator (Sonics & Materials, Inc). The lysate obtained after sonication was centrifuged at 24000*g* for 45 min at 4 °C. The supernatant was collected and heated at 70 °C for 30 min to precipitate most of the *E. coli* proteins and centrifuged at 24000*g* for 45 min at 4 °C. The supernatant was then treated with 0.01% PEI to precipitate nucleic acids and centrifuged at 24000*g* for 45 min at 4 °C. A Q-Sepharose anion exchange chromatography column connected to a Akta Basic HPLC from GE Healthcare Life Sciences, UK was equilibrated using buffer A (20 mM Tris–HCl, pH 8, 10% glycerol, 0.1 mM EDTA, 2 mM DTT, and 0.1 mM PMSF), and the supernatant from PEI treatment was loaded on to the column. Thereafter, the column was washed with three column volumes of buffer A. The protein was then eluted using a linear gradient of 0 to 30% of 1 M NaCl in buffer A. The protein fractions collected were loaded on 12% SDS-PAGE and stained using Coomassie Brilliant Blue. The fractions containing pure protein were pooled, dialyzed, and concentrated using Amicon Ultra 10 kDa cut-off filters. The concentration of the purified protein was estimated by the Bradford method using bovine serum albumin as standard, divided into aliquots, and stored at −80 °C.

### Design of MS experiments

This study characterized the purified proteins of WT and variants of MjGATase through MS. The MS analysis was carried out in two ways; first, intact protein mass analysis and second, MS/MS analysis of in-gel tryptic peptides. The experimental parameters are provided in detail below. The results from these experiments report on the presence or absence of specific modifications in WT and mutants of MjGATase.

### Mass spectrometric analysis of intact proteins

The MjGATase proteins were diluted to 1 mg ml^−1^ in water and dialyzed against water using a 10 kDa cut-off membrane. The protein was further diluted to 0.1 to 0.2 mg ml^−1^ in water with 0.1% formic acid. Then, 10 to 30 pmol of the dialyzed protein sample was injected on to a C8 reverse phase column (Thermo Fisher Scientific, 2.1 mm diameter, length: 100 mm, 5 μm particle size, 175 Å pore size). Mass spectral analysis was carried out on a Q Exactive HF mass spectrometer (Thermo Fisher Scientific) equipped with a Thermo Scientific Dionex Ultimate 3000 UHPLC system at the in-house Central Instrumentation Facility, Molecular Biology and Genetics Unit, JNCASR. The protein was eluted using water containing 0.1% formic acid (solvent A) and acetonitrile containing 0.1% formic acid (solvent B) as the solvent system at a flow rate of 0.3 ml min^−1^. The HPLC run conditions were as follows: equilibration at 10% B for 1 min, gradient from 10% B to 80% B over 6 min, isocratic at 80% B for 1 min, gradient of 80% B to 95% B in 1 min, isocratic at 95% B for 2 min, back to 10% B in 1 min, and finally equilibrate at 10% B for 3 min. The column temperature was set to 40 °C. Full MS scans were acquired at a resolution of 120,000 at m/z 200, scan range of 500 to 2000 m/z, and automatic gain control target set to 1e6 with maximum ion injection time of 100 ms. The mass spectrometer was operated at a spray voltage of 3.8 kV, capillary temperature of 320 °C, auxiliary gas heater temperature of 200 °C, and S-lens radio frequency (RF) level value set at 80. The flow rates of sheath gas and auxiliary gas were set to 20 and 10, respectively.

MjGATase_E108Q protein was diluted to 0.1 mg ml^−1^ in 50% methanol and MS-grade water with 0.1% formic acid. The mass spectrum was acquired by direct injection of the protein through a Hamilton syringe in the positive ion mode. The Q Exactive HF mass spectrometer was operated with a scan range of 400 to 2000 *m/z*, resolution of 120,000 at *m/z* 200, spray voltage of 3.5 kV, capillary temperature of 275 °C, probe heater temperature of 200 °C, and S-lens RF level value at 80. The flow rates of sheath gas and auxiliary gas were set to 25 and 10, respectively. The ion injection time and ion target value were set at a threshold of 200 ms and 3e6, respectively.

Data collection and visualization were carried out with Xcalibur v4.1 software and Qual browser (Thermo Fisher Scientific). Deconvolution of the spectra was carried out using Biopharma finder v3.2 software (Thermo Fisher Scientific). Xtract algorithm was used for deconvolution as our data were isotopically resolved. The source spectra were generated by using “average over selected retention time” method. A relative abundance threshold cutoff of 10 to 15% above the noise level of the spectrum was set during deconvolution.

### In-gel trypsin digestion

The protocol followed was as described earlier (38) and derived from that developed by Mann and co-workers (75). Briefly, 30 μg of the protein was run on 12% SDS-PAGE and stained with Coomassie Brilliant Blue. The stained gel piece carrying the protein band was cut into small pieces, washed with water, and destained using a 1:1 ratio of methanol and 50 mM Tris–HCl, pH 7.0. The gel pieces were dehydrated by first using a 1:1 ratio of acetonitrile and 50 mM Tris–HCl, pH 7.0, followed by 100% acetonitrile for 30 s. The liquid was removed, and the gel pieces were dried in speed-vac and rehydrated in 25 mM DTT in 50 mM Tris–HCl, pH 7.0, and incubated at 56 °C for 20 min. Thereafter, the gel pieces were washed with water, dehydrated, and dried as described above. The gel pieces were rehydrated with buffer containing 500 ng of trypsin in 50 mM Tris–HCl, pH 7.0, such that it covered the gel pieces and incubated at 37 °C for 12 h. The tryptic peptides were extracted twice with 50% acetonitrile and 5% formic acid. The extracted peptides were pooled together and dried in a SpeedVac. The dried peptides were dissolved in 2% acetonitrile and 0.1% formic acid and desalted using C18 spin columns (Thermo Fisher Scientific). The fractions eluted from the C18 spin columns were dried using a SpeedVac.

### Mass spectrometric analysis of tryptic peptides

The dried peptides were dissolved in 20 μl of MS-grade water with 0.1% formic acid. Mass spectral analyses were carried out on a Q Exactive HF mass spectrometer (Thermo Fisher Scientific) equipped with an Easy nanoLC 1200 (Thermo Fisher Scientific). Data were acquired in positive ion mode using the higher-energy collision-induced dissociation fragmentation method. Easy-Spray column (Thermo Fisher Scientific), PepMap RSLC C18 of 2 μm particle size, 100 Å pore size, 75 μm inner diameter, and 25 cm length along with a C18 guard column (Acclaim PepMap100), of 3 μm particle size, 100 Å pore size, 75 μm inner diameter, and 2 cm in length was used. Column temperature was set to 40 °C and autosampler temperature was set to 4 °C. Water with 0.1% formic acid as solvent A and 80% acetonitrile with 0.1% formic acid as solvent B was used. The flow rate was set at 300 nl min^−1^.

The peptides of the MjGATase_SNN_109_ and mutants were eluted using the following steps; a gradient of 5% B to 25% B in 65 min, isocratic at 25% B for 5 min followed by a gradient of 25% B to 95% B over 15 min, isocratic at 95% B for 10 min, gradient of 95% B to 5% B in 2 min, and finally equilibration at 5% B for 8 min. The mass spectrometer was operated in a data-dependent mode with a scan range of 300 to 3800 *m/z* and a resolution of 120,000 at *m/z* 200 Th. The top 15 abundant ions were fragmented by higher-energy collision-induced dissociation at a normalized collision energy of 30 and the ddMS2 was acquired at a resolution of 15,000 at *m/z* 200 Th, a scan range of 200 to 2000 *m/z*, and dynamic exclusion set to 5 s. The ion injection time and ion target value were set at a threshold of 50 ms and 3e6 for MS scans and 50 ms and 1e5 for MS/MS scans. A nanospray ion source was used with a spray voltage of 1.7 kV, capillary temperature of 275 °C, and S-lens RF level set to 55. Data acquisition was carried out using Xcalibur software v4.1 (Thermo Fisher Scientific).

Data analyses were done with Proteome Discoverer (PD) v.2.3 and v.2.4 software and Qual browser of Xcalibur software v4.1 (Thermo Fisher Scientific). The search engine, Sequest HT in PD, was used with an input of MjGATase sequence into the protein database. For the mutants, the FASTA files of MjGATase sequence containing the variant amino acid was provided as input. The following search parameters were used: semitrypsin as enzyme, precursor mass tolerance set to 10 ppm, and fragment mass tolerance of 0.05 Da. The maximum missed cleavage sites allowed for tryptic peptides was set to 2. Dynamic modifications of oxidation at Met (+15.995 Da), ammonia-loss at Asn (−17.027 Da), and deamidation at Asn/Gln (+0.984 Da) leading to the conversion of -CONH_2_ to -COOH were included. Dynamic modification of acetylation (+42.011 Da) at N terminus was also provided. The XCorr score from Sequest HT was used to assign confidence for each PSM. The protein coverage of MjGATase_SNN_109_ and eight mutants from MS/MS analysis of tryptic peptides was in the range of 98 to 100%.

### Crystallisation and data collection

Crystallization of the mutant proteins of MjGATase, D110P, D110_K151L and K151L_Y158F was set up under a 1:1 mixture of silicon and paraffin oil using the micro-batch method. The conditions from commercially available kits of Hampton Research and Molecular Dimensions were used to set up the crystallization screens and the trays were kept in a room maintained at 25 °C. The crystals were obtained within 7 to 10 days. MjGATase_D110P crystals were obtained in 0.2 M zinc acetate dihydrate with 20% PEG 3350, pH 6.4., MjGATase_D110V_K151L crystals were obtained in 0.1 M MES, pH 6.5, 0.2 M ammonium sulfate, 30% PEG monomethyl ether 5000, and MjGATase_K151L_Y158F crystals were obtained in 0.1 M MES, pH 6.5, 0.2 M ammonium sulfate, 30% PEG monomethyl ether 5000. The crystal, prior to diffraction, was presoaked in the cryoprotectant containing 20% glycerol for MjGATase_D110P and MjGATase_D110V_K151L, and 10% glycerol for MjGATase_K151L_Y158F before mounting it on the goniometer head.

X-ray diffraction data were collected at the X-ray facility at the Molecular Biophysics Unit, Indian Institute of Science, Bangalore. X-ray diffraction data on all crystals were collected using a Rigaku RU200 X-ray diffractometer. This was equipped with a rotating anode-type light source with an osmic mirror that gives a monochromatic light source of wavelength 1.54179 Å.

### Structure determination and refinement

Processing of the diffraction images was carried out using iMOSFLM (76). SCALA module and PHASER module of CCP4 were used for scaling and phasing, respectively (77, 78, 79). The molecular replacement method was used to obtain structure solutions using MjGATase_SNN_109_ (PDB ID: 7D40) structure as the model. The refinement of the structures was carried out using the REFMAC module of CCP4 (80) and manual refinement was carried out using COOT software (81). The structures are available online on the RCSB-PDB website with the PDB IDs as mentioned in Table 1. All structure analyses were carried out using PyMOL (https://www.pymol.org/) and superposition of protein structures was carried out using default settings of outlier rejection (5 cycles, cutoff 2).

### Thermal denaturation

The denaturation temperature was determined by absorbance measurements using a UV spectrophotometer. Solutions of MjGATase_SNN_109_ and mutant D110P, D110V_K151L, and K151L_Y158F proteins of MjGATase at 10 μM protein concentration in 20 mM Tris, pH 7.4, were incubated for 30 min at different temperatures of 25, 70, 75, 80, 85, and 90 °C in a dry bath. At 100 °C, the proteins were incubated for 15 min. The proteins were later centrifuged at 16000*g* for 15 min to pellet down the precipitated protein. The supernatant containing the remaining soluble protein was collected and the absorbance spectrum was acquired from 200 to 400 nm on a UV-visible spectrophotometer (Shimadzu, UV-1780). The spectrum of the buffer that was treated in the same manner as the protein samples was also acquired and subtracted from the protein spectrum. The T_m_ was then estimated from the plot of the absorbance at 280 nm against the temperature.

Protein thermal unfolding of MjGATase_SNN_109_ and the variants K107L, E108L, E137L, K151L, and Y158F were analyzed by CD as described earlier (38, 40). Ellipticity of the protein solution at 220 nm was recorded using a cuvette of 0.1 cm path length with a temperature ramp of 0.5 °C min^−1^ and a temperature range of 70 °C to 100 °C. Similarly, for D110P and the two double mutants, the thermal unfolding was monitored from 60 to 100 °C. These measurements were carried out using a Jasco J-810 spectropolarimeter (Jasco Corporation) equipped with a Peltier heating system at a protein concentration of 10 μM in 5 mM Tris–HCl, pH 7.4. The spectrum of the buffer components was subtracted from the spectra of all the protein samples.

### Rate of denaturation

To estimate the rate of denaturation of the MjGATase_SNN_109_ and mutant D110V_K151L and K151L_Y158F proteins of MjGATase, the absorbance spectra were recorded for solutions of the proteins heated at a fixed temperature for varying times. Solutions of WT and mutant proteins at a protein concentration of 10 μM in 20 mM Tris, pH 7.4, were initially equilibrated at 70 °C for 7 min in a dry bath and ramped up to 80 °C. At 80 °C, protein solutions were incubated for 10, 15, 30, 45, 60, 90, 120, and 180 min. Thereafter, the solutions were centrifuged at 16000*g* for 15 min to pellet down the precipitated protein. The soluble protein in the supernatant was collected and the absorbance spectrum was acquired from 200 to 400 nm on a UV-visible spectrophotometer (Shimadzu, UV-1780). The protein spectra were corrected for the background by subtracting the spectrum of buffer components. The rate of denaturation was then estimated from the plot of the absorbance at 280 nm against the incubation time.

### Estimating the increase in SNN population by lowering the pH of protein

MjGATase_D110V_K151L protein at 10 μM concentration in 20 mM ammonium formate, pH 3.0, was incubated at 60 °C for 16.5 h and subjected to LC-MS analysis. The same protein aliquot stored in −80 °C was diluted to 10 μM concentration in 20 mM ammonium formate, pH 3.0, and without prior incubation, LC-MS analysis was carried out. This served as the 0^th^ time point of the experiment. After incubation, the protein sample was centrifuged at 16000*g* for 10 min at 4 °C before loading on the column. Five picomoles of the protein sample was injected on to a reverse phase C8 column (Thermo Fisher Scientific, 2.1 mm diameter, 100 mm length, 5 μm particle size, and 175 Å pore size). Mass spectral analysis was carried out on a Q Exactive HF mass spectrometer (Thermo Fisher Scientific) connected to a Thermo Scientific Dionex Ultimate 3000 UHPLC system. The column temperature was maintained at 30 °C. The injected protein was eluted using water with 0.1% formic acid (solvent A) and acetonitrile with 0.1% formic acid (solvent B) as the solvent systems and a flow rate of 0.3 ml min^−1^. The gradient used, MS settings, HESI source settings, data collection, visualization, and deconvolution are as mentioned above in mass spectrometric analysis of the intact proteins.

### Activity of MjGATase

MjGATase and MjATPPase (UniProt ID: Q58531) subunits associate to form functional GMPS. Hence, the activity of MjGATase was measured by coupling it with MjATPPase as reported (38). Briefly, XMP to GMP formation was monitored using a UV-visible spectrophotometer (Shimadzu, UV-1780) by measuring the continuous decrease in absorbance at 290 nm. The reaction mix of total volume 500 μl consisted of 90 mM Hepes, pH 7.0, 3 mM ATP, 200 mM XMP, 20 mM MgCl_2_, and 5 mM glutamine. The assay temperature was maintained at 70 °C. The reaction was initiated by adding a mixture of MjGATase and MjATPPase. XMP (ε_290_ = 4800 M^−1^ cm^−1^) to GMP (ε_290_ = 3300 M^−1^ cm^−1^) conversion, which leads to a drop in Δ ε_290_ of 1500 M^−1^ cm^−1^ was used for the estimation of GMP concentration (82). The protein concentration of 3.48 μg of MjATPPase and 2.1 μg of MjGATase was used.

### Computational methodology

The MjGATase_SNN_109_ contains the PTM, SNN, at the 109th position (PDB ID: 7D40) (38, 40), a product of Asn deamidation and cyclization of the side chain CG with the n+1 backbone amide nitrogen. To understand the mechanism of this SNN formation, we needed to generate the initial configuration or the reactant state (containing Asn) through *in silico* mutation. We used chain A from PDB 7D40 and Gaussview (56) for this purpose (See Supplemental text). The ϕ and ψ values for SNN are in the fourth quadrant of the Ramachandran map, which is a disallowed region for L-amino acids. Hence, CMD was carried out in two steps: first, using a united atom force field GROMOS54A7 (83, 84) and second with AMBER99sb-star-ILDNforce field (57, 58, 85). By directly using AMBER force field alone, we could not obtain the right set of backbone dihedral angles for Asn109. Upon employing GROMOS force field, backbone dihedral angles for the residue 109 were sampled in the allowed region of the Ramachandran plot. The last frame from the GROMOS trajectory was used as the initial conformation to continue with AMBER family of forcefield. Along with AMBER99sb-star-ILDN for protein, TIP3P (86) parameters were used for water molecules.

We used GROMACS software ((87) http://www.gromacs.org) for CMD simulations and CP2K software (88) patched with PLUMED (89, 90) for QM/MM MD simulations. The protein was placed in a periodic cubic box with 20 Å periodic image distance (box length 72 Å) and solvated using the GROMACS solvation module. Protonation states of ionizable residues were set through the GROMACS default program. Three sodium ions were added to neutralize the complete system. Periodic boundary conditions were applied in all three directions. For maintaining temperature and pressure, we used the Bussi-Donadio-Parrinello thermostat (91) and the Parrinello-Rahman barostat (92), respectively. Time constants for temperature coupling at 298 K were 0.5 ps and 100 fs for CMD and QM/MM MD simulations, respectively. Pressure coupling was used only for CMD runs during equilibration. Pressure was maintained at 1 bar isotropically (isothermal compressibility: 4.5 × 10^−5^ bar^−1^) with a 1.0 ps time constant. Real space cutoffs for the Lennard-Jones and Coulomb interactions were set to 10 Å and 14 Å for AMBER and GROMOS, respectively. Lorentz-Berthelot mixing rules were used to obtain the LJ parameters between two atom types. The particle Mesh Ewald (93) method with an interpolation order of 4 and a relative tolerance of 10^−5^ was used to calculate the electrostatic interactions for distances above 10 (or 14) Å for CMD calculations in GROMACS. A Smooth particle Mesh Ewald (94) method with an interpolation order of 6 and a relative tolerance of 10^−6^ was used to calculate the electrostatic interactions for distances above 10 Å for MM calculations in CP2K. For running the QM/MM MD simulations in CP2K, QUICKSTEP (95) and FIST modules were used. The quantum region was described at the DFT level with the Perdew-Burke-Ernzerhof functional (96). A double-ζ valence-polarized basis set was used along with Goedecker-Teter-Hutter pseudopotentials (97) (DZVPGTH-Perdew-Burke-Ernzerhof). The plane wave cutoff was set at 300 Ry, and DFT-D3 dispersion correction was applied (98). The MM region was described using the same force field used for CMD simulations. The reaction was studied using an enhanced sampling technique (nontempered metadynamics (99) with two reaction coordinates (*i.e.* collective variables) and the electrostatic embedding framework to account for the electrostatic interactions between QM and MM regions. Our collective variables (along with the upper wall bias) are shown in Figure 8*B* in the reactant state generated from the multistep multiscale equilibration. Each of the two collective variables was a combination (using PLUMED function COMBINE) of two coordination numbers (using PLUMED function COORDINATION, see Supplemental text section for the same). CV_1_ is the combination of the number of contacts between "atom 1 and atom 2" and "atom 2 and atom 3". CV_2_ is the combination of the number of contacts between "atom 3 and atom 4" and "atom 1 and atom 4". R_0_ value used for the two sets of coordination numbers defining CV_1_, that is, (1, 2) and (2, 3) was 1.34 Å each, respectively. And the same was 1.00 Å each for defining CV_2_, *i.e.*, (3, 4) and (1, 4). Default values were used for other parameters. So, as per the definition, the values for the combined coordination numbers or collective variables of the reactant and product states were (−0.5, −0.5) and (+0.5, +0.5), respectively (Fig. 8*A*). In addition, we used an upper wall bias (using PLUMED function UPPER WALL) between the two nitrogen atoms (side chain amide-N of Asn109 and side chain amino-N of Lys151, labeled as atom 3 and atom 5, respectively) at 5.5 Å with a force constant of 200 kcal/mol (remaining parameters with default values). For the metadynamics bias, the sigma values for CV_1_ and CV_2_ were 0.005 and 0.006, respectively, and 0.59 kcal/mol hills were added every 100 fs. During analysis, the criteria for successful hydrogen bonding were fixed at a donor-acceptor distance of <3.5 Å and a donor-H-acceptor angle of >140°. The bin width for histogramming is chosen as 0.01 Å.

Time steps for integrating the equations of motion for CMD and QM/MM MD simulations were set as 1 and 0.5 fs, respectively. Leap-frog algorithm was used for solving equations of motion. Position restraining was applied using a harmonic potential with a force constant of 10^3^ kJmol^−1^ nm^−2^ for the nonhydrogen atoms. All bonds were constrained using LINCS with order 4 and a warn angle of 30^◦^.

The general protocol for equilibration using both forcefields for CMD consisted of four steps: a) energy minimization of water molecules (position restraining the protein), b) energy minimization of protein atoms (position restraining water), c) equilibration at constant volume and temperature (NVT ensemble) by raising the temperature from 0 to 298 K over 500 ps and keeping at 298 K for the next 500 ps, and d) equilibration at constant temperature and pressure (NPT ensemble) at 298 K and 1 atm, respectively, for 1 ns. This was followed by the production run in NVT ensemble at 298 K for 100 ns. The nonhydrogen protein coordinates from the 100th ns frame of the GROMOS trajectory was used as the initial conformation to continue with AMBER force field. From the AMBER production trajectory, 1 arbitrary protein conformation was used to initiate QM/MM MD simulations (Fig. 8*B*).

We have carried out one CMD simulation with MjGATase_SNN_109_ structure as well. This consisted of three steps of equilibration—i) energy minimization, ii) equilibration at constant volume and temperature (NVT ensemble), and iii) equilibration at constant temperature and pressure (NPT ensemble) which is followed by a production run in NVT. The same GROMOS parameter set was used here as well.

Treating the complete system with QM description is highly computationally expensive for such a large system; hence, only some residues relevant to the reaction (suggested from experiments) were considered at the QM level and others were treated at the MM level. Hence, the QM level contained residues 109 (Asn), 110 (Asp), 151 (Lys), and 158 (Tyr). To make sure that we were not cutting through any polarizable bonds, a part of residue 108 (backbone carbonyl of Glu) were included in the QM region. The total number of quantum atoms was thus 61 (Fig. 8*B*) that included four link atoms (hydrogen atoms) capping the QM region. The quantum box spanned 15 Å in three directions. QM/MM equilibration was done in three steps: a) energy minimization with mechanical embedding, b) energy minimization with electrostatic embedding, and c) equilibration at constant volume and temperature (NVT ensemble) with electrostatic embedding.

A summary of simulations is tabulated in Table S4. The complete system is shown in Figure 8*A*.

Note: Simulation input files are available at https://github.com/Oishika-1/QM-MM_MjGATase_Succinimide. Simulation data files and analysis scripts would be shared upon request. The reaction VMD movie has been provided at https://drive.google.com/drive/u/0/folders/1dmEbYcDjfDQnFDsQDvq_IthEq4mt9vIa.

## Data availability

All data related to this article are contained within the main text and supporting information.

The MS data have been deposited to the ProteomeXchange Consortium *via* the PRIDE (100) partner repository with the dataset identifier PXD075095 and PXD075155 (https://doi.org/10.6019/PXD075155).

The structures with PDB IDs: 7YC6, 8GR1, and 8GR3 are available on the RCSB-PDB website.

The model, MjGATase_ASN_109_, used for QM/MM MD metadynamics is available in ModelArchive at https://modelarchive.org/doi/10.5452/ma-vn1er.

## Supporting information

This article contains supporting information.

## Conflict of interest

The authors declare that they have no conflicts of interest with the contents of this article.
